# Letter to Dr. Bowling

**Published:** 1855-07

**Authors:** Emma Willard


*Extract of a Letter from Mrs. Emma Willard, to Dr. Bowling.*

There has been of late *in my practice*, (for I constantly practice Æripathy for my own health; and sometimes among my friends,) one case, among a set of cases less remarkable, which ought to be recorded. In crossing the Atlantic last June, in the steamer Pacific, some of the passengers soon began to suffer, as is usual, from sea-sickness. And when a lady of my acquaintance came out of her state-room suffering, and having that kind of bluish purple about her face which indicated deficient respiration, I advised her to try such forcible breathing as would throw all the air out of her lungs, and draw plenty of fresh air in. She and some others practiced as I directed them, and although they said it seemed impossible that that should be the cause, yet they certainly felt relieved. About the fourth day of our voyage, as I was walking the deck with a Mr. Campbell—now of New York, but recently of Montreal—a Scotch gentleman of high respectability—he told me that his partner in business, Mr. Martine, had been so deadly sea-sick, that he had as yet kept his berth, not having left his state-room at all since we left New York. While he was yet speaking, he turned his eyes to the stair-way, where was just heaving in sight a gentleman perfectly pale, and apparently weak, supported by a lady. "There," said Mr. Campbell, "there he is—that is Martine with his wife. This fine morning has tempted him forth." On enquiry, Mr. Martine said that he still suffered intensely. He had an intense head-ache, as well as a nausea and a feeling of oppression; he should soon be obliged to take to his berth again Could you have faith, said I, that a woman could do you any good? He at once quite gallantly declared that he could have faith in me, and would implicitly follow any directions which I would give him; and Mr. Campbell and Mr. Martine went his surety that he would. Mr. Martine and Mr. Campbell both considered this case so remarkable that it ought to be reported in some Medical Journal; and both promised to give their testimony in writing. I believe the three witnesses mentioned are now in New York. Then we all took an extreme corner of the deck. I placed my patient facing the breeze. Now, said I, you are to believe that your lungs are at this moment filled with a heavy irrespirable gas, and your breathing is so feeble that you do not throw it out, and you are in the condition of a person suffering from the fumes of charcoal. (His face had the same ghastly violet blue, pinched, and half collapsed appearance.) And what I want you to do (and I showed him as well as described) is to get this heavy gas out of your lungs. Make your chest as small as possible, by stooping; drawing down your ribs, and pressing your arms to your sides; throw out the air by a violent and long continued *exhaling*—blowing it from your mouth as if engaged in blowing up a fire. Then change, make a long and forcible inhalation, opening your chest to its fullest dimensions by standing erect, and raising your arms from the shoulders. Mr. Martine perfectly comprehended my instructions before he began to practice them; and when he did, I wish that you had been there to note the change which came over him within one or two minutes. He had not breathed over three or four of those forced, long breaths, before the pale face, changing as we looked upon it, assumed the glow of health. The lips changed from violet blue to coral red, and the cheeks (for my patient had as fine a complexion as as any body) changed from no color to rosy red; his eyes were bright and his heart was cheered, and he exclaimed, "Why, I feel well; I never felt better in my life!" He then exercised briskly by walking the deck, after which he went to his dinner, and from thenceforward was regular at his meals, and was no more confined to his room during the voyage. If he felt the sea-sickness at any time coming on, he pushed for the air, and forcibly ventilated his lungs, and then he was well again. Now, I am not going to assert that sea-sickness can always be cured in this way—but when the patient has been some time sick, and is in the condition in which I found Mr. Martine, I know not why others should not be cured by the same process.*

You are aware, I dare say, that Dr. Cartwright has not, or had not some time ago, been able to see the agency of caloric in the process by which the blood is moved; but that he has in this respect a theory of his own which teaches that the cause of motion is life in the blood. If I understand Dr. Dowler, he maintains that the blood does work, which has formerly been attributed to the nerves. Although I have felt that the agency of Caloric is essential both to the proof and the utility of my theory, and in fact the very theory itself, I cannot see that my views in this respect, (as stated and maintained by Dr. Washington,) if allowed to be correct, are at all in the way of Dr. Cartwright's and Dr. Dowler's making discoveries in the track they are upon; which seems to be something different and beyond the mere moving of the blood. It is one thing to carry a loaf of bread and a vial of medicine, and quite another the use which shall be made of them when carried to their destination. The power which moves them may be the same, while their effects though taken into the same stomach, may be altogether different. All I pretend to, refers to the carrying process, with the common known effects of the blood, as circulated or not

* The able surgeon of the Pacific became, during the voyage, a thorough believer in the truth of the theory by which this cure was effected. Mr. and Mrs. Martine and Mr. Campbell all being intelligent witnesses, left in this case no shadow of doubt as to the fact of the cure related, of which he had already known something by Dr. Cartwright's alligator experiments.

circulated. The question concerning the life of the blood seems as I said, beyond; and it is one from which important results may spring. I know a case intimately well, of a lady, who, twenty years ago, in making a long walk in a funeral procession where she was unable to stop, felt a very acute pain, like a sudden cut on the outer part of her left limb just above the knee, she at the same time apprehended some lesion in the interior of the limb; but she was obliged to continue walking for some distance farther, and the result was a permanent injury; coldness in the limb, pain, and a slight, gradual diminution in size. The whole left side was in some measure affected. A physician was consulted, who supposed the cause of the difficulty was in the nervous system, some paralytic symptoms having supervened. But I believe the lady's difficulty to be in the sanguinous system. I thought that some derangement of the blood vessels had deprived the limb of its proper quantity of blood. About eight years ago, while attending a large party where she was obliged to stand, this lady was (after a feeling of excessive fatigue) suddenly seized with a pain in the part of the limb affected so agonizing, that it made her instantly pale and faint. The result was that after this, she began to find her limb graduully becoming restored to warmth, natural feeling and size. She believed that at the time she felt the last sudden anguish, the obstruction which had existed to the blood's right course gave way, and left it more to its natural flow. She accustomed herself to practice exercise and full breathing, generally before an open window, and then when the blood in her system was flowing freely, she seated herself on a sofa in a position to cramp the right side, and leave the left free, the left limb extended on the sofa, and the whole left side higher than the right. By this course she obtained more blood upon the left side. She is now relieved from a series of symptoms, so long painful and threatening her with paralysis. If in this case the difficulty was in the stoppage of the blood, and the relief in the restoration of its course, and the means otherwise used to force it from the right side to the left, does not the case favor Dr. Cartwright's theory, that the life is in the blood more than in the nerves? or is it that the nerves lie dormant until excited by the presence of the blood? These are certainly very important enquiries.

